# Trap Engineering-Based Optimization via Polyetherimide with Molecular Semiconductor for Capacitive Energy Storage at High Temperatures

**DOI:** 10.3390/polym17172294

**Published:** 2025-08-25

**Authors:** Dingqu Liu, Hao Chen, Lihe Guo, Hongfei Li, Haiping Xu

**Affiliations:** 1 Shanghai Engineering Research Center of Advanced Thermal Functional Materials, Shanghai Polytechnic University, Shanghai 201209, China; 20241518066@sspu.edu.cn (D.L.); 20231518005@sspu.edu.cn (H.C.); lhguo@sspu.edu.cn (L.G.); 2 Shanghai Key Laboratory of Engineering Materials Application and Evaluation, Shanghai Research Institute of Materials, Shanghai 200437, China

**Keywords:** dielectric, trap energy level, high temperature, energy storage

## Abstract

Polyetherimide (PEI)/molecular semiconductor-based all-organic dielectric composites have garnered significant attention due to their exceptional energy storage performance at elevated temperatures. In this work, the high-electron-affinity semiconductor 5,6,12,13-tetrachloro-2,9-bis(2-ethylhexyl)anthra[2,1,9-def:6,5,10-d′e′f]diisoquinoline-1,3,8,10(2H,9H)-tetraone (TCEHAQ) is employed as a filler to enhance the dielectric energy storage performance of PEI. It is believed that TCEHAQ can immobilize electrons and reduce charge transport in dielectric composites. The results demonstrate that the breakdown strength of PEI with only 0.5 wt% of TCEHAQ increased from 450 MV/m to 600 MV/m at room temperature, while the maximum discharge energy density (*U_d_*) reached 5.99 J/cm^3^, and the discharge efficiency (*η*) was 96.5%. Meanwhile, the breakdown strength of the 0.5 wt% TCEHAQ/PEI blend at 150 °C was 500 MV/m, and the maximum *U_d_* and *η* were 3.68 J/cm^3^ and 81.0%, respectively. This is a straightforward and effective method for fabricating large-area, high-quality dielectric energy storage films suitable for use in harsh environments.

## 1. Introduction

Dielectric capacitors with high power density are critical components of advanced electrical and electronic systems [1]. Polymer dielectric materials, as essential components of dielectric capacitors, are widely utilized due to their flexibility, ease of processing, unique self-healing properties, as well as their superior breakdown electric field strength compared to ceramic dielectrics [2]. However, it has become increasingly challenging for current polymer dielectric materials to meet the growing demand in harsh conditions, particularly at extreme temperatures [3]. For instance, biaxially oriented polypropylene (BOPP), which is widely used in commercial applications, can only function at temperatures below 105 °C and exhibits a charging/discharging efficiency of merely 60% at 400 MV/m at 120 °C [4,5]. Meanwhile, the lower dielectric constant (2.2) of BOPP limits the obtainment of high-energy storage densities, and the injection, excitation, and transport of carriers at high temperatures and high electric fields lead to a significant decline in energy density [6,7].

The development of high-performance dielectric materials that can function steadily at high temperatures has emerged as the most significant research focus. Engineering polymers with high glass transition temperatures (*T_g_*), including polyimide (PI), polybenzimidazole (PBI), polyether ether ketone (PEEK), and polyetherimide (PEI), are continuously being manufactured and utilized. PI, one of the most widely used high-temperature engineering plastics, is an ideal material for high-temperature film capacitors because of its outstanding mechanical strength, high *T_g_*, high-temperature resistance, and superior insulating properties. However, its performance at elevated temperatures is significantly inferior to that at room temperature; the discharge energy density (*U_d_*) of PI is only 0.44 J/cm^3^ at 150 °C [8]. Polyetherimide (PEI), with flexible ether bonds (-O-) embedded in the PI backbone, has garnered significant attention for research into its energy storage properties under high temperatures [9]. PEI, with a *T_g_* exceeding 200 °C, is considered an effective candidate for high-temperature dielectric applications, where the highly conjugated π-π aromatic ring structure of PEI promotes the excitation, injection, and transport of carriers under electric and thermal stresses, resulting in a noticeable increase in conductivity [10,11]. This conduction loss is usually attributed to various mechanisms, including Schottky emission and hopping conduction [12,13]. Electron hopping conduction in polymer dielectrics is typically governed by the insulating properties of the polymer itself. In this process, thermally activated charge carriers transition from one trap site to another via the tunneling effect [14]. To reduce leakage current surges and associated high conductive losses, researchers have attempted to introduce nanomaterials such as silica and alumina to enhance the dielectric constant and thermal stability [15]. By optimizing the filler type and content, carrier migration can be suppressed to some extent, and conduction losses can be reduced, thereby improving the energy storage efficiency of the materials under extreme conditions [16,17]. However, comparatively low trap energy levels tend to arise because of the low *ε_r_* and outstanding insulating qualities of BN, Al_2_O_3_, and other materials [18,19]. More significantly, high-surface-energy inorganic nanoparticles are normally unstable and have a propensity to aggregate inside the polymer matrix, which facilitates carrier hopping and ultimately results in breakdown [20]. To efficiently trap and scatter carriers, deeper trap energy levels must be established within the polymer [21]. It is evident that certain molecular semiconductors, such as PCBM, ITIC, and DPDI, which possess high electron affinities, have been incorporated into polymer matrices. These semiconductors have been demonstrated to generate deeper electron traps, thereby effectively reducing leakage currents. However, the high manufacturing cost of molecular semiconductors has limited their commercial mass production and application [22].

In this work, we synthesized a novel semiconductor, 5,6,12,13-tetrachloro-2,9-bis(2-ethylhexyl)anthra[2,1,9-def:6,5,10-d′e′f′]diisoquinoline-1,3,8,10(2H,9H)-tetraone (TCEHAQ), in which four Cl atoms were incorporated on the perylene ring. The Cl atoms have high electron affinity, which increases the electron affinity of the material. Then, TCEHAQ was doped into PEI to prepare all-organic composites. The high electron affinity of TCEHAQ effectively establishes deep electron traps in PEI to capture free electrons, which improves the dielectric constant and effectively suppresses the leakage current at high temperatures. To verify these hypotheses, an investigation was conducted into the dielectric performance, polarization mechanism, and energy storage properties of TCEHAQ/PEI blends.

## 2. Experimental Methods

### 2.1. Materials

Polyetherimide (PEI, *ρ* = 1.27 g/cm^3^) was obtained from PolyK Technologies, State College, PA, USA; the molecular semiconductor TCEHAQ was synthesized in-house. N-Methyl pyrrolidone (NMP, Shanghai Titan Technology Corporation, Shanghai, China) and PEI were used directly, without pretreatment. 2-Ethylhexylamine (99%), 1,6,7,12-tetrachloroperylene tetracarboxylic acid dianhydride (>95%), and propionic acid (99%) were commercially available from Adamas.

### 2.2. Preparation of TCEHAQ/PEI Polymer Blends

**Figure 1:**
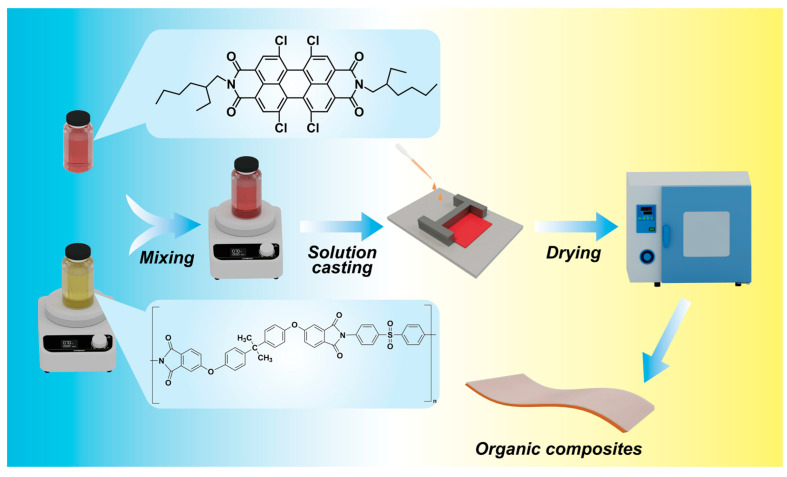
Preparation of TCEHAQ/PEI blended films.

The preparation procedure for the TCEHAQ/PEI blends is illustrated in Figure 1. According to Figure 1, the TCEHAQ/PEI blend film was prepared, demonstrating the outstanding film-forming qualities of the polymer mixture. The all-organic composite films were fabricated using the solution casting method. The semiconductor powder required for each gradient was first dissolved in NMP separately. Then, the high-temperature-resistant polymer PEI was dissolved in NMP and stirred for 2 h. The NMP solution of the molecular semiconductor was mixed with the polymer solution, stirred for 5 min, and then sonicated for 60 min using a tip-type sonicator (150 W) to obtain a series of mixed solutions, with concentrations of 0.25 wt%, 0.5 wt%, 0.75 wt%, and 1 wt%. The solutions were then drop-cast onto a clean glass slide. The cast film was dried for 12 h at 80 °C in a drying oven to eliminate the solvent, then heated to 125 °C and 150 °C for 1 h each, and finally dried at 200 °C for 12 h to further remove any residual solvent. After immersion in deionized water for 30 s, the films were peeled off from the glass substrate and dried in a vacuum oven at 100 °C for 12 h. Typically, the films utilized for electrical characterization had thicknesses ranging from 8 to 12 µm.

### 2.3. Preparation Process of Organic Molecular Semiconductor TCEHAQ

Under a nitrogen atmosphere, 1,6,7,12-tetrachloroperylene tetracarboxylic acid dianhydride (2.12 g, 4 mmol), 2-ethylhexylamine (1.55 g, 12 mmol), and 85 mL of propionic acid were added to a 150 mL reaction vial, stirred until the samples were well dispersed, and then the reaction was refluxed for 40 h. At the end of the reaction, the mixture was cooled to room temperature, and the reaction solution was slowly poured into saturated ammonium chloride, extracted with THF three times, and the organic phase was dried with anhydrous MgSO_4_. Using DCM/CH_3_OH (150:1–125:1) as the eluent, 3.19 g of red product was obtained after separation on a silica gel column with an 87% yield. ^1^H NMR (500 MHz, DMSO, ppm): δ 7.67 (s, 4H, pery), 3 (s, 2H, NCH_2_CH), 3.43–3.01 (m, 12H, CH_3_), 2 (s, 2H, CH), 1.24 (m, 16H, CH_2_).

### 2.4. Characterization

The crystal structures of the composite films were analyzed using X-ray diffraction measurements (XRD, Bruker, Saarbrücken, Germany) on an X-ray polycrystalline diffraction spectrometer. A thermogravimetric analyzer (TG, Netzsch STA 449 F3, NETZSCH—Geratebau GmbH, Bavaria, Germany) was used to study the thermal stability properties of the films, and the dispersion of the semiconductors in solution was observed by transmission electron microscopy (TEM, FEI Talos F200X G2, FEI Company, Hillsboro, OR, USA). Copper electrodes were deposited on both surfaces of the film by the vacuum coating method. The dielectric constant and dielectric loss were measured using a broadband dielectric impedance spectrometer (Concept 80, Novocontrol GmbH, Montabaur Germany). Polarization and electric field (*P*-*E*) hysteresis loops of the nanocomposite films immersed in silicone oil were characterized using a ferroelectric analyzer (Radiant Multiferroelectric II 100V, Radiant Technologies Inc., Washington, DC, USA) at room temperature.

## 3. Results and Discussion

### 3.1. Structure of the Molecular Semiconductor

**Figure 2:**
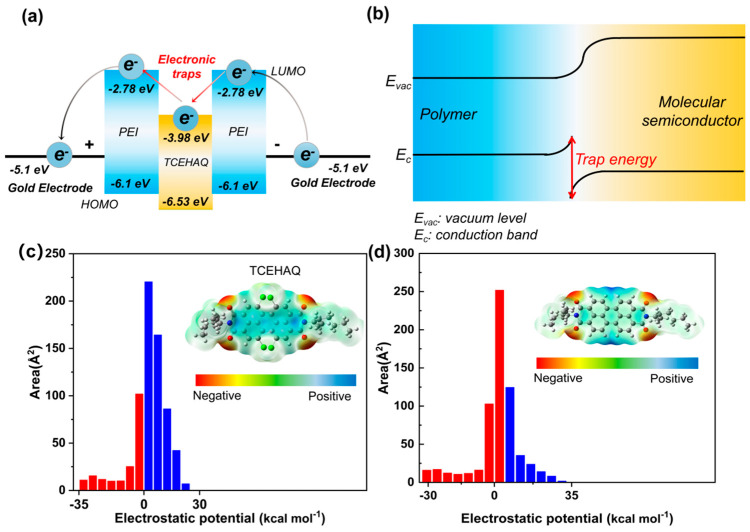
(**a**) Simplified energy band diagram of possible electron migration in TCEHAQ/PEI. (**b**) Schematic diagram of the trap energy generated by TCEHAQ in PEI. (**c**,**d**) Electrostatic potential distribution and area percentage in each electrostatic potential range of molecular semiconductors with and without Cl.

The molecular structure of the semiconductor TCEHAQ, blended with PEI, is shown in Figure 1. TCEHAQ has obvious symmetry, with the perylene ring at the center as the axis of symmetry and substituents on both sides. The introduction of Cl atoms into the perylene ring, where chlorine acts as electron-withdrawing group, reduces the π-electron density of the aromatic skeleton, and modulates the conjugated electronic structure and energy level distribution of the molecule, thereby enhancing the electron affinity of the material. Density Functional Theory (DFT) was used to analyze the distribution of electrostatic potentials of TCEHAQ with and without Cl atoms, and the LUMO energy level was directly related to the strength of its electron-capturing ability. As can be seen from Figure 2a,b, TCEHAQ lowers the LUMO energy level of PEI by 1.2 eV, which indicates that TCEHAQ improves PEI′s ability to capture electrons [23]. As shown in Figure 2c,d, it is consistent with our preliminary analysis. The strong electron-withdrawing effect of the chlorine atom causes the electrostatic potential of TCEHAQ to be predominantly positive (highlighted in blue). The presence of Cl atoms with greater electron affinity attracts electrons and creates deeper carrier traps, thus improving breakdown.

### 3.2. Morphology of Polymer Blends

**Figure 3:**
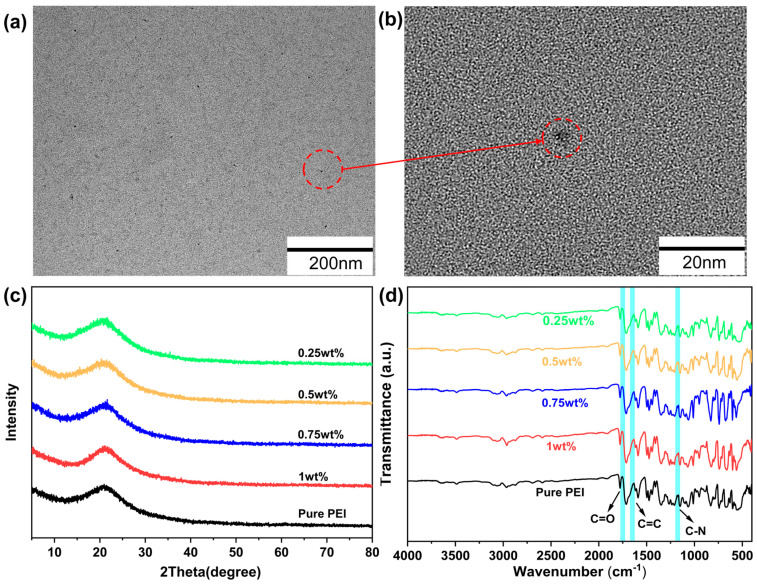
(**a**) Low-magnification TEM image and (**b**) high-magnification spherical aberration-corrected TEM image of the TCEHAQ clusters. (**c**) The XRD patterns of pure PEI and the composite films loaded with different contents of TCEHAQ. (**d**) The FTIR spectra of the composite films loaded with different contents of TCEHAQ.

To investigate the compatibility of PEI and semiconductors in the blended polymers, the dispersion of semiconductors in solution was analyzed using TEM. As shown in Figure 3a,b, the semiconductor nanoparticles achieved ideal uniform dispersion in the polyetherimide matrix. This shows that PEI has good compatibility with TCEHAQ and can form high-quality films. The inhomogeneous dispersion of TCEHAQ leads to phase-separated interfaces, which can trigger localized charge accumulation. The interfacial buildup results in electric field distortion, increasing the leakage current and leading to a decrease in breakdown strength. XRD and FTIR spectra of TCEHAQ/PEI hybrid films with different contents and TCEHAQ are shown in Figure 3c,d, respectively. As shown in Figure 3c, the XRD diffractogram clearly shows that both the pure PEI film and the TCEHAQ/PEI blend film have a single broad peak corresponding to the distinctive form of the PEI peak. As shown in Figure 3d, the chemical functional group structure of the TCEHAQ/PEI composite polymer material was analyzed using FTIR spectroscopy. For PEI, the characteristic peaks shown in the spectra include the carbonyl group (C=O) at 1740 cm^−1^, the carbon–carbon double bond (C=C) at 1620 cm^−1^, and the carbon–nitrogen bond (C-N) at 1144 cm^−1^. The characteristic peaks of molecular semiconductors are hardly visible in the TCEHAQ/PEI composite dielectric material, which can be attributed to the ultra-low concentration of the molecular semiconductors, as well as the overlap of their major characteristic peaks with those of PEI. The positions and shapes of the characteristic peaks are essentially identical for pure PEI and the TCEHAQ/PEI composites. This demonstrates that TCEHAQ does not chemically interact with PEI to form new chemical bonds.

### 3.3. Dielectric Properties of Polymer Blends

**Figure 4:**
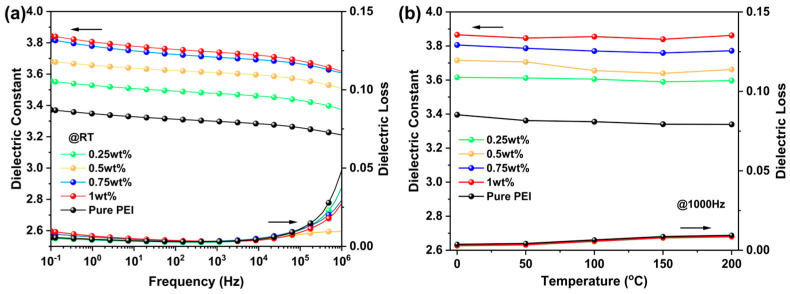
(**a**) *ε_r_* and tanδ of TCEHAQ/PEI composites as functions of frequency. (**b**) *ε_r_* and tanδ of TCEHAQ/PEI composites as functions of temperature.

Figure 4a illustrates the relationship between *ε_r_* and tanδ of TCEHAQ/PEI polymer blends with varying TCEHAQ content at room temperature as a function of frequency. The *ε_r_* of pure PEI at 1 kHz is 3.3, and tanδ is 0.05. Additionally, the dielectric properties of pure PEI film are virtually independent of frequency from 10^−1^ Hz to 10^6^ Hz. The influences of *ε_r_* and tanδ on TCEHAQ/PEI blends are minimal within the frequency range of 10^−1^ to 10^6^ Hz. This indicates that the introduction of TCEHAQ did not significantly affect the dielectric stability of PEI, but rather moderately enhanced its dielectric constant, further optimizing the comprehensive performance of the material. This phenomenon is primarily caused by interfacial polarization. On one hand, when the mass fraction of TCEHAQ is low, no significant phase separation occurs in the polymer blend film, due to the good compatibility between TCEHAQ and PEI. On the other hand, the organic molecular semiconductor TCEHAQ has a larger electron affinity than PEI and can capture injected charges through stronger electrostatic attraction [24]. As a result of the introduction of TCEHAQ, the *ε_r_* of the blended film is further enhanced by the increased interface polarization strength of TCEHAQ/PEI polymer blends [25]. The reduction in dielectric loss is due to the molecular affinity of TCEHAQ and its ability to form deep traps in the polymer matrix that binds free electrons, as well as the incorporation of organic molecular semiconductors that can modulate carrier mobility and establish a more stable charge transport pathway. When carriers are captured by the trap, they can form controlled ohmic conduction through the influence of an electric field, rather than disordered migration. This ordered conduction minimizes charge accumulation and local electric field distortion within the dielectric, thereby reducing Joule heating and dielectric losses caused by space charge polarization. In addition, the glass transition temperature (*T_g_*) of the doped polymer composites increased compared to the undoped system, as demonstrated by the DSC curve (Figure S1). This is attributed to the incorporation of rigid structural units (e.g., perylene rings) into the molecular semiconductors, which restricts the high-temperature movement of the polymer chain segments. Meanwhile, the TG curve (Figure S2) shows that the thermal weight loss temperature of the doped material increases due to the strong electron affinity of the molecular semiconductor, which stabilizes the charge interactions between the polymer chains. Additionally, carrier trapping reduces the accumulation of Joule heat and delays thermal degradation. The TG and DSC analyses collectively demonstrate that the organic molecular semiconductor suppresses high-temperature leakage conduction through deep traps, and the rigid structural units increase the *T_g_*, enhancing the thermal stability of the polymer matrix. Furthermore, the leakage current density of the blends was significantly reduced, further confirming their excellent insulating properties. The findings of this study indicate that the TCEHAQ/PEI blends have considerable prospects for use in high-frequency and high-temperature environments, making them an optimal material choice for the fabrication of high-performance electronic devices.

In addition, the temperature reliability of the dielectric characteristics of the polymer blends was investigated by evaluating the *ε_r_* and tanδ of the PEI and TCEHAQ/PEI blend samples at elevated temperatures (10^3^ Hz, as shown in Figure 4b). As the temperature increased from 25 °C to 200 °C, the *ε_r_* of the pure PEI film decreased, from 3.36 to 3.33, and the tanδ gradually increased, from 0.002 to 0.008. The *ε_r_* of the 0.5 wt% TCEHAQ/PEI composite decreased, from 3.71 to 3.66, and the tanδ slowly increased, from 0.002 to 0.008. Similar to pure PEI, the *ε_r_* of TCEHAQ mixtures with different contents has good temperature stability, with almost no change in *ε_r_* in the temperature range of 25–200 °C and 10^3^ Hz. The high glass transition temperature of PEI is the primary factor contributing to this result. Meanwhile, the incorporation of a minimal quantity of TCEHAQ did not reduce the thermal stability of the TCEHAQ/PEI polymer blends. Overall, the results of the dielectric tests showed that the TCEHAQ/PEI polymer blends exhibit excellent dielectric properties with temperature and frequency dependence.

### 3.4. Breakdown Strength and Polarization of Polymer Blends

In addition to the dielectric constant, breakdown strength is a critical factor in the energy storage performance of dielectric capacitors. Accordingly, the two-parameter Weibull statistical distribution method was used to examine the *ε_r_* of the polymer blends and to analyze the breakdown strength.

(1) $$P(E)=1-\exp\left(-\right.({\frac{E}{E_{b}})}^{\beta })\;$$

**Figure 5:**
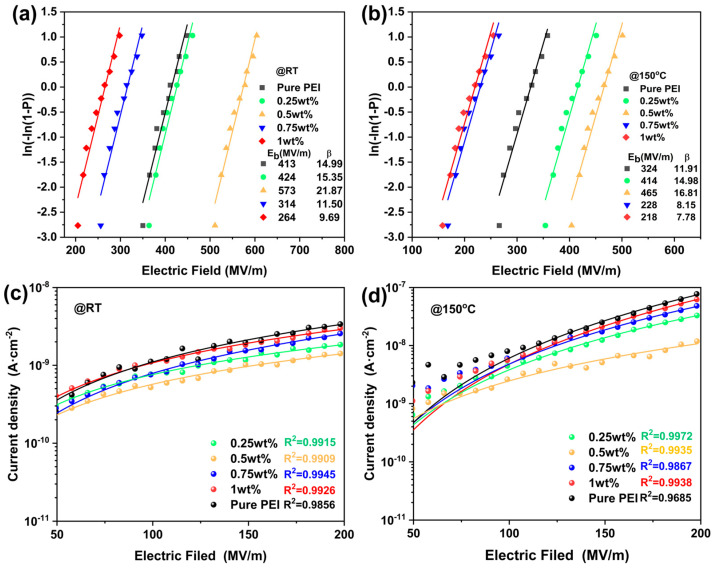
Breakdown strength and leakage current of TCEHAQ/PEI composites at high temperatures. (**a**,**b**) Weibull distribution of dielectric breakdown strength at room temperature and 150 °C; (**c**,**d**) hopping conduction model fitting for leakage current at room temperature and 150 °C.

In which *P*(*E*) is the cumulative failure probability, and *E* is the actual measured electric field strength. *E_b_* denotes the Weibull breakdown strength, and *β* is a shape parameter that reflects the reliability of the measured electric field. The larger the value of *β*, the higher the reliability of the experimental data [26,27]. Figure 5a shows the *E_b_* of the TCEHAQ/PEI polymer blends with different TCEHAQ mass fractions. The *E_b_* of the TCEHAQ/PEI polymer blends first increased as the TCEHAQ mass fraction rose. Subsequently, a decline was observed in conjunction with the sustained increase in the TCEHAQ mass fraction. As shown in Figure 5a, the *E_b_* value of pure PEI at room temperature was 413 MV/m, and the *E_b_* value of the TCEHAQ/PEI mixture, containing 0.5 wt% of TCEHAQ, was 573 MV/m. When the TCEHAQ content increased to 1 wt%, the breakdown field strength decreased, from 573 MV/m to 264 MV/m, falling below that of pure PEI. As shown in Figure 5b, TCEHAQ/PEI polymer blends with 0.5 wt% TCEHAQ exhibit an *E_b_* value of 465 MV/m at 150 °C, while those containing 1 wt% TCEHAQ show a reduction in *E_b_* value, from 465 MV/m to 218 MV/m. The change in the *E_b_* of the all-organic polymer blend material results from TCEHAQ trapping electrons and reducing the leakage current [27,28]. An optimal balance was achieved with a TCEHAQ content of 0.5 wt%, which enhanced the breakdown strength of the hybrid films. This is attributed to the high electron affinity of the molecular semiconductor TCEHAQ, which can trap free electrons to form deep trap energy levels and, thus, inhibit their migratory behavior under strong electric fields or high temperatures. This trapping mechanism reduces the leakage current of dielectric composites. In pure PEI, the lack of an organic semiconductor with high electron affinity to trap free electrons facilitates the formation of conductive pathways within the polymer, leading to high leakage current density and low breakdown strength in PEI. On the contrary, Figure 5c,d clearly show that the leakage current density of the TCEHAQ/PEI blend is lower than that of PEI. At lower TCEHAQ content, the molecular semiconductor TCEHAQ, with high electron affinity in the polymer co-dopant, traps a large number of free electrons, which is able to block the long-distance migration of the charge, thereby reducing the leakage current density and enhancing the *E_b_*. In particular, the 0.5 wt% TCEHAQ/PEI polymer blend exhibits the lowest leakage current density. This accounts for the highest *E_b_* observed in the 0.5 wt% TCEHAQ/PEI polymer blend. As additional molecular semiconductor TCEHAQ is incorporated, the aggregation of TCEHAQ molecules becomes increasingly pronounced, resulting in a corresponding reduction in the average intermolecular distance. This has been shown to result in an increased probability of electron carrier conduction. Consequently, the TCEHAQ/PEI polymer blend is more susceptible to breakdown under high electric fields. As a result, the *E_b_* of the TCEHAQ/PEI polymer blend with 1 wt% TCEHAQ is reduced in comparison to its blend with a lower content of TCEHAQ.

### 3.5. Energy Storage Properties of Polymer Blends

As posited by the hysteresis loop theory, it is feasible to obtain the discharge energy density (*U_d_*) of the capacitor by using the following, Equation (2):

(2) $$U_{d}=\int EdD$$

Regarding the charging and discharging efficiency (*η*) of the capacitor, it is derived from the ratio of *U_d_* to the charging energy density (*U_c_*).

(3) $$\eta =\frac{U_{d}}{U_{c}}\cdot$$

**Figure 6:**
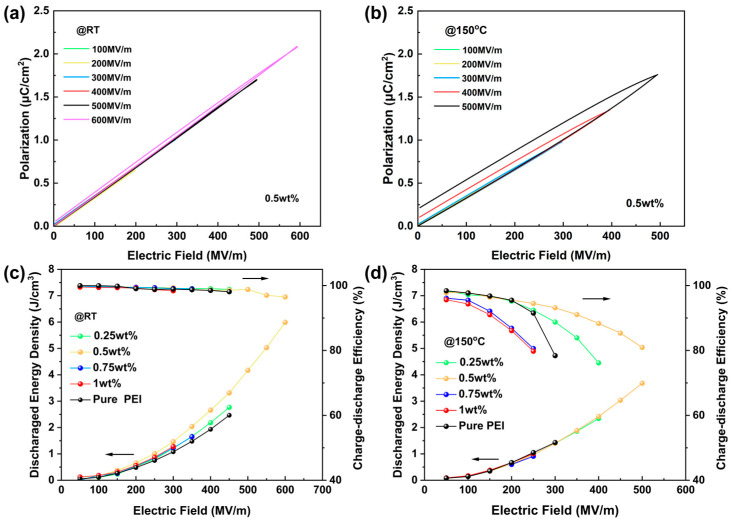
The *P*-*E* loops of the films at (**a**) room temperature and (**b**) 150 °C. *U_d_* and *η* of the films at (**c**) room temperature and (**d**) 150 °C.

In the above equation, *E* denotes the applied electric field. Polarization–electric field (*P*-*E*) loops for all polymer films are presented in Figure 6a,b and Figures S4–S7. These were measured by the ferroelectric analyzer at both room temperature and 150 °C. It is evident that the high electron mobility and interfacial polarization of TCEHAQ are the primary factors contributing to this phenomenon. It has been demonstrated that the degree of polarization in the composite films is significantly higher than that of pure PEI when an equivalent electric field is applied. Evidently, the maximum *E* and the maximum polarization (*Pmax*) of the composite films first increase, and then decrease, with an increase in the content of TCEHAQ. For the purpose of illustration, the maximum *E* of 0.5 wt% TCEHAQ/PEI (600 MV/m) is 1.3-fold higher than that of pure PEI (450 MV/m). Meanwhile, the *U_d_* values of all TCEHAQ/PEI polymer blend films exceeded those of pure PEI films, thus indicating that a beneficial effect is achieved by the addition of molecular semiconducting TCEHAQ fillers into the PEI matrix, resulting in increased *U_d_* values and the maintenance of high *η* values.

The parameters of interest in this study are *U_d_* and *η*, which are critical for evaluating dielectric polymers. *P*-*E* loops were used to calculate *U_d_* and *η.* The values of *U_d_* and *η* for all samples at room temperature, and at the elevated temperature of 150 °C, are presented in Figure 6c,d. The addition of TCEHAQ has minimal impact on the polarization of PEI membranes, and the energy density is almost constant under low electric fields. It was observed that pure PEI exhibited accelerated performance degradation at elevated temperatures and high electric field conditions; in contrast, the composite materials demonstrated the ability to maintain over 90% energy efficiency at higher field strengths. As a case in point, at a temperature of 150 °C, the 0.5 wt% TCEHAQ/PEI sample achieved the highest *U_d_* of 3.68 J/cm^3^ (*η* = 81.0%), representing a 1.9-fold increase compared to 1.88 J/cm^3^ (*η* = 52.1%) for pure PEI. The *U_d_* of 0.5 wt% TCEHAQ/PEI at room temperature under *η* > 90% reached 5.99 J/cm^3^ (*E* = 600 MV/m), which was a 2.4-fold increase compared to 2.46 J/cm^3^ for pure PEI (*E* = 450 MV/m). These results indicate that TCEHAQ effectively excites and traps the charge within the matrix, reducing the leakage current density at high temperatures and under high electric fields. This reduces the likelihood of the leakage current evolving into breakdown current, which improves the *E_b_* of PEI-based composites. Consequently, the TCEHAQ/PEI composite films exhibit superior efficiency and reliable energy storage performance under extreme conditions.

## 4. Conclusions

In summary, the present study has investigated the dielectric and energy storage properties of blends comprising an all-organic polymer with molecular semiconductor TCEHAQ as a filler and linear dielectric PEI as a matrix. The all-organic polymer TCEHAQ/PEI blends were prepared using a cost-effective solution casting method. The findings demonstrated that the 0.5 wt% polymer blend exhibited the maximum *U_d_* of 5.99 J/cm^3^ with *η* of 96.5% at 600 MV/m at room temperature. Meanwhile, the 0.5 wt% polymer blend had the largest *U_d_* of 3.68 J/cm^3^ with *η* of 81.0% at 500 MV/m at 150 °C. This is due to the strong interfacial polarization resulting from the accumulation of space charge at the interface between the TCEHAQ filler and the PEI matrix. The organic molecule semiconducting TCEHAQ with high electron affinity traps free charge and inhibits long-range charge migration. In addition, PEI has a high glass transition temperature and exhibits exceptional high-temperature resistance, thereby imparting the TCEHAQ/PEI polymer blends with outstanding dielectric and energy storage properties. These blends maintain excellent performance in high-temperature environments, further validating their potential for application under extreme conditions.

## Data Availability

The raw data supporting the conclusions of this article will be made available by the authors on request.

## Supplementary Materials

The following supporting information can be downloaded at: https://www.mdpi.com/article/10.3390/polym17172294/s1, Figure S1. DSC curves of the composite films loaded with different content of TCEHAQ; Figure S2. TG curves of the composite films loaded with different content of TCEHAQ; Figure S3. Histogram of *T_g_* versus *T_d_* for pure PEI and composite film with 0.5 wt% content; Figure S4. *P*-*E* cycles of pure PEI films at (a) room temperature and (b) 150 °C; Figure S5. *P*-*E* cycles of 0.25 wt% TCEHAQ films at (a) room temperature and (b) 150 °C; Figure S6. *P*-*E* cycles of 0.75 wt% TCEHAQ films at (a) room temperature and (b) 150 °C; Figure S7. *P*-*E* cycles of 1 wt% TCEHAQ films at (a) room temperature and (b) 150 °C; Figure S8. DSC curve and TG curve of TCEHAQ; Figure S9. The FTIR spectra of TCEHAQ; Figure S10. ^1^H NMR spectra of monomer TCEHA.
