# Supplementary material for: Trap Engineering-Based Optimization via Polyetherimide with Molecular Semiconductor for Capacitive Energy Storage at High Temperatures
Source: Polymers (Basel). 2025 Aug 25;17(17):2294. doi: 10.3390/polym17172294 (PMC12430510; doi:10.3390/polym17172294)
Supplement: Supplementary file 1 [file polymers-17-02294-s001.zip › polymers-3761823-supplementary.pdf]

Supporting Information

Trap Engineering-based Optimization via Polyetherimide with Molecular Semiconductor for capacitive energy storage at high temperatures

Dingqu Liu [1], Hao Chen [1], Lihe Guo [1], Hongfei Li [1,2,*], and Haiping Xu [1,*]

[1]Shanghai Engineering Research Center of Advanced Thermal Functional Materials, Shanghai Polytechnic University, Shanghai 201209, China

[2] Shanghai Key Laboratory of Engineering Materials Application and Evaluation, Shanghai Research Institute of Materials, Shanghai 200437, China

[*] Correspondence: hfli@sspu.edu.cn (Hongfei Li), hpxu@sspu.edu.cn (Haiping Xu)

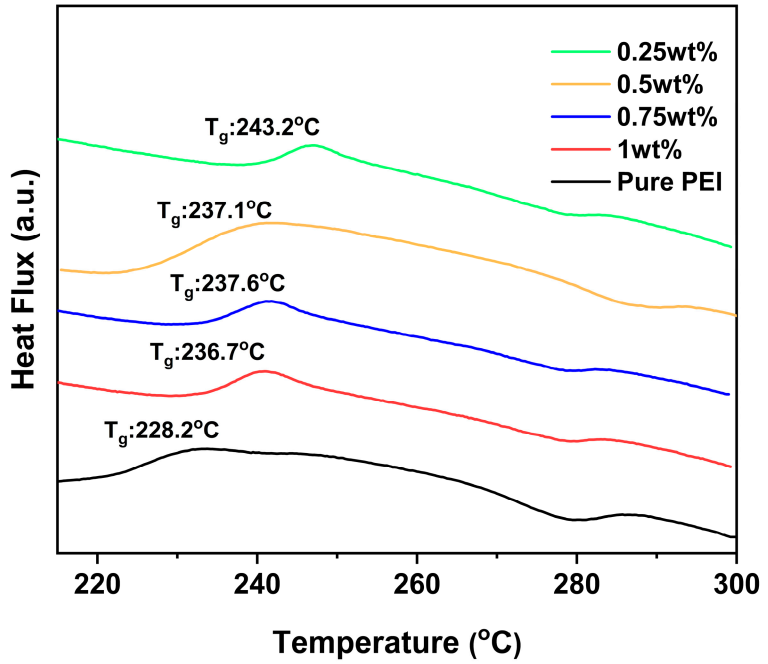

Figure S1. DSC curves of the composite films loaded with different content of TCEHAQ.

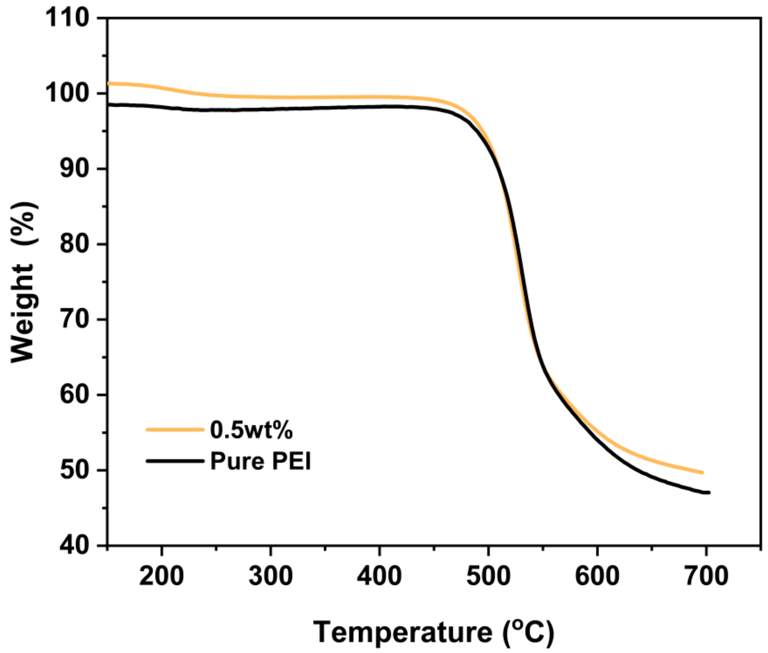

Figure S2.TG curves of the composite films loaded with different content of TCEHAQ.

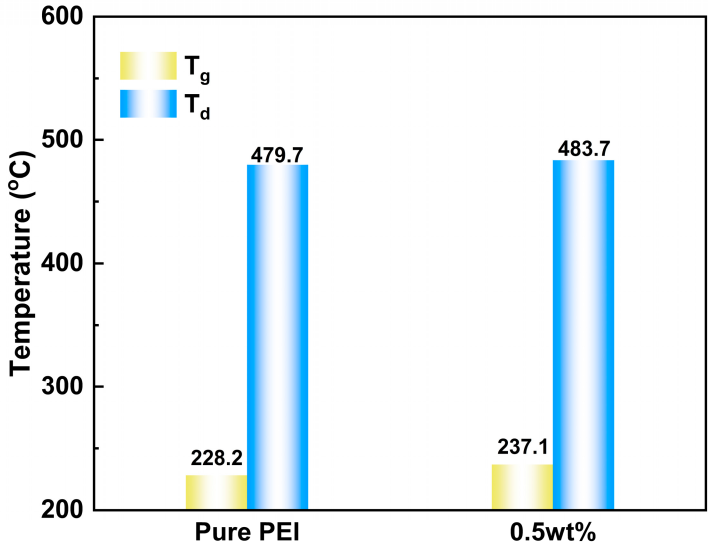

Figure S3. Histogram of T_g versus T_d for pure PEI and composite film with 0.5 wt% content.

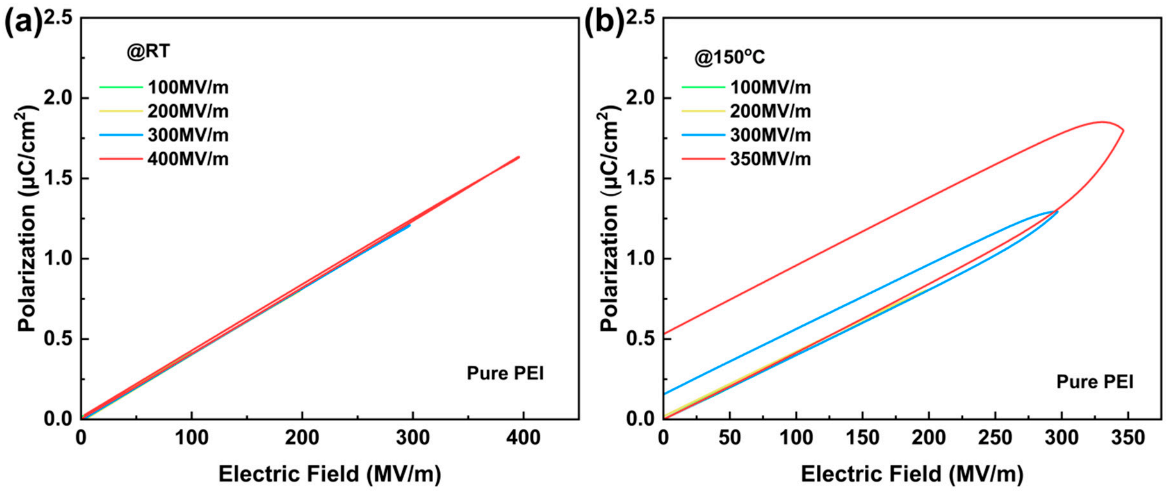

Figure S4. P-E cycles of pure PEI films at a) room temperature and b) 150 °C.

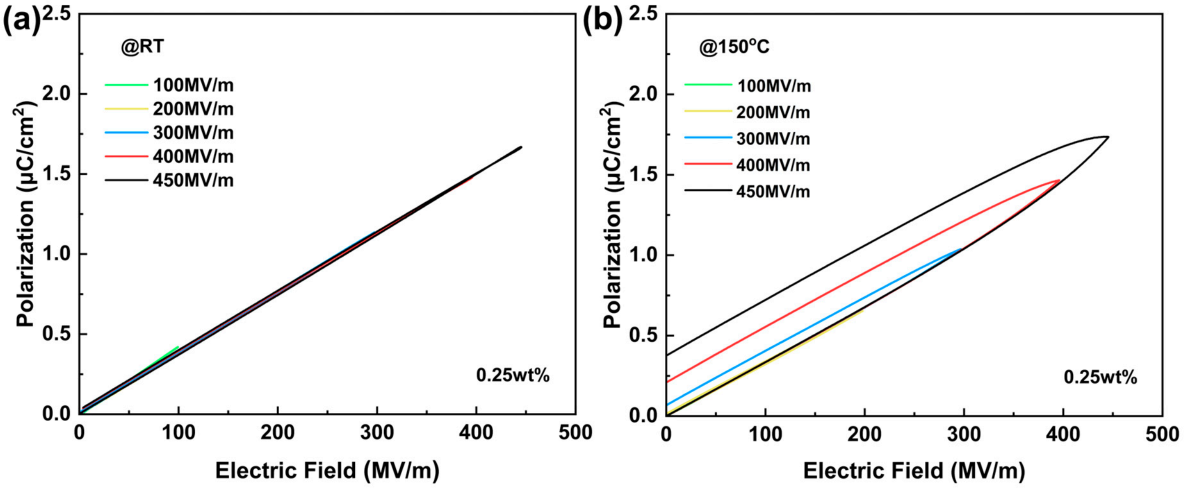

Figure S5. P-E cycles of 0.25wt% TCEHAQ films at a) room temperature and b) 150 °C.

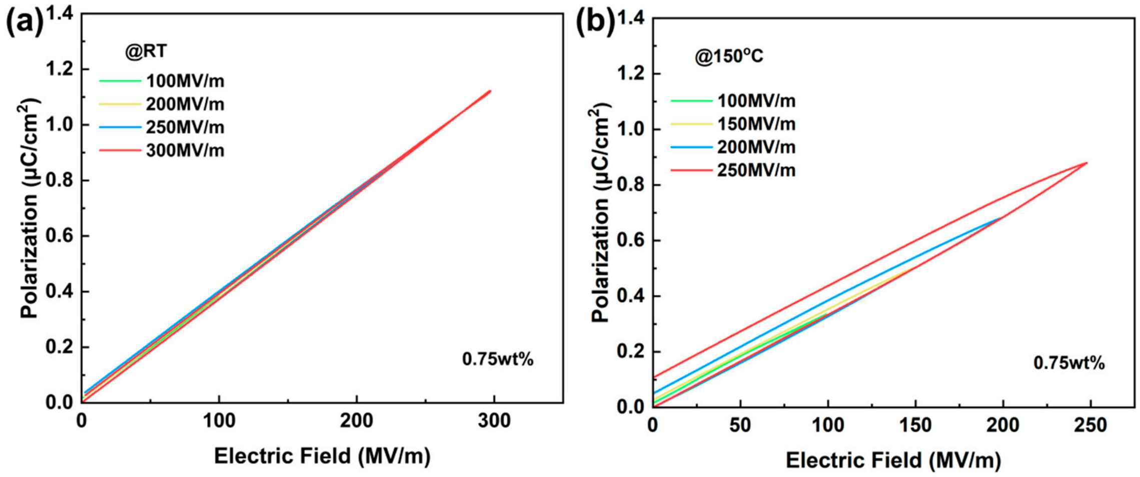

Figure S6. P-E cycles of 0.75wt% TCEHAQ films at a) room temperature and b) 150 °C.

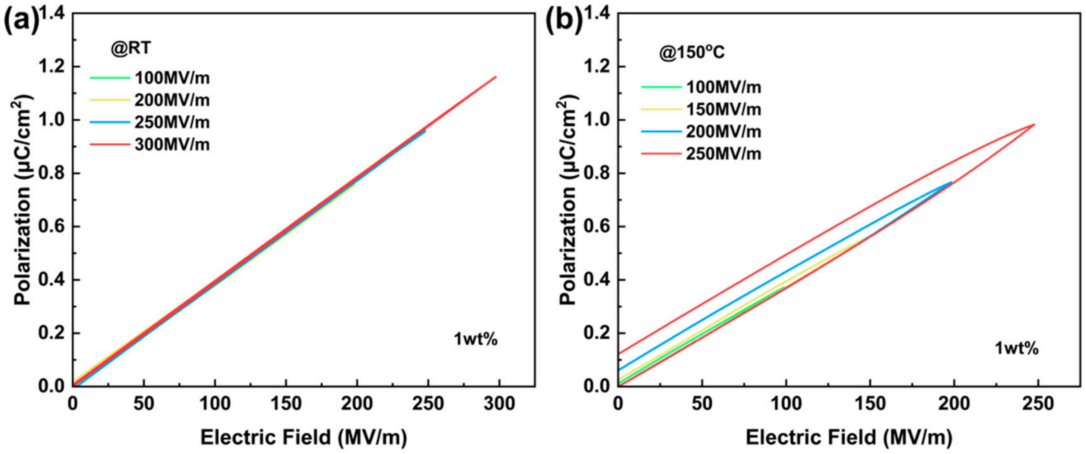

Figure S7. P-E cycles of 1wt% TCEHAQ films at a) room temperature and b) 150 °C.

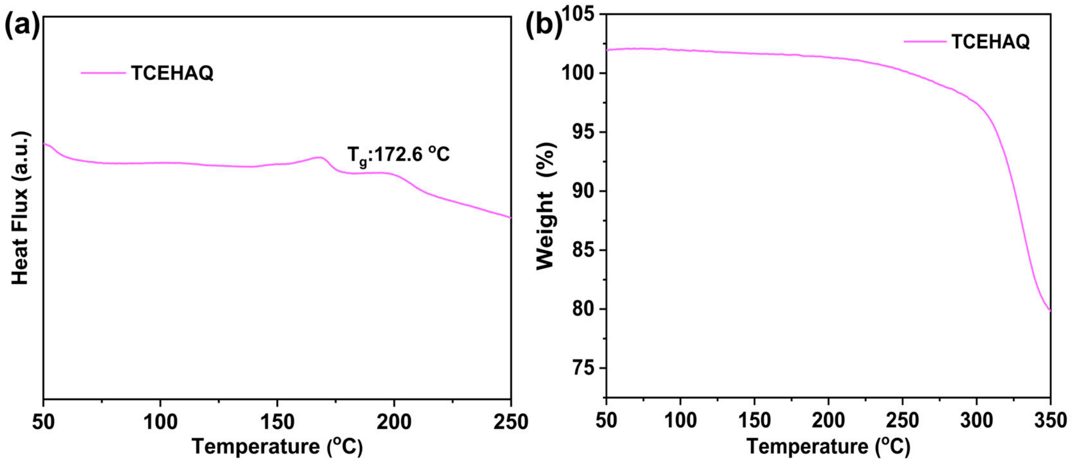

Figure S8. DSC curve and TG curve of TCEHAQ.

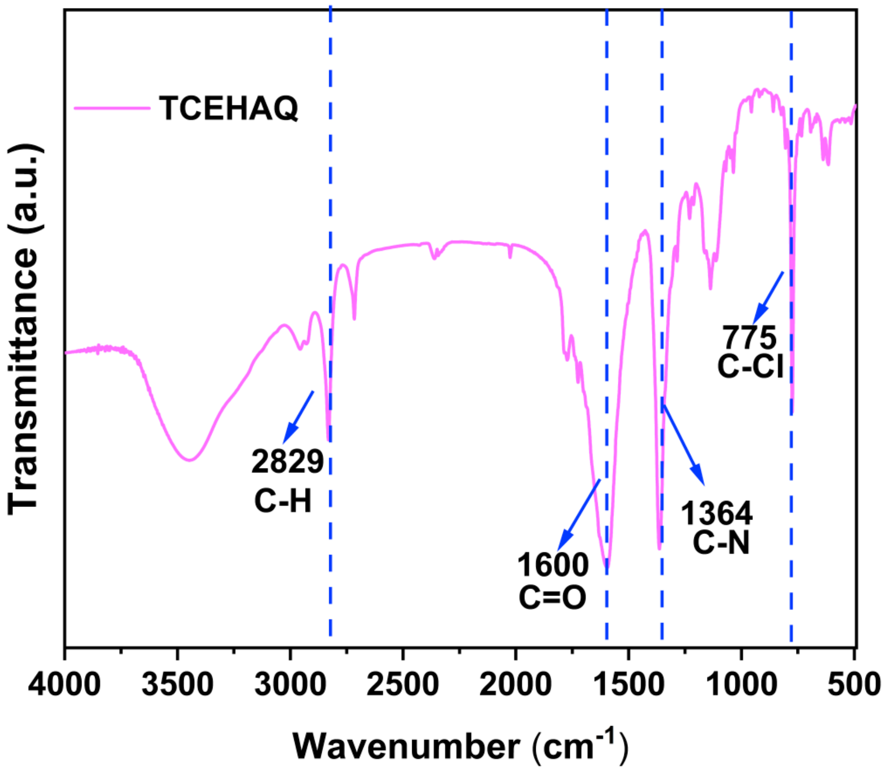

Figure S9. The FTIR spectra of TCEHAQ.

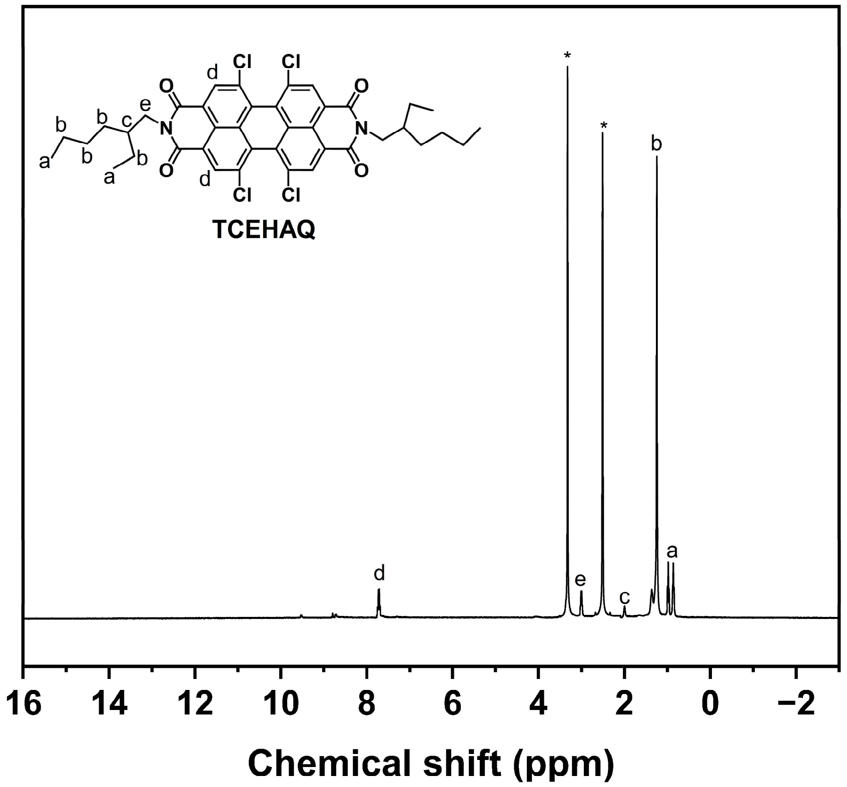

Figure S10. ^{1}H NMR spectra of TCEHAQ.
